# Identifying sleep disturbance- and fatigue-related factors of poor health-related quality of life in patients with advanced ovarian cancer

**DOI:** 10.1186/s41687-026-01040-1

**Published:** 2026-03-23

**Authors:** Abigail Newell, Erica E. Fortune, Elif Andac-Jones, Maria B. Gonzalo, Elizabeth A. Szamreta

**Affiliations:** 1 Cancer Support Community, Research & Training Institute, Washington, DC USA; 2 Merck & Co., Inc, Outcomes Research, Oncology, Rahway, NJ USA

**Keywords:** Ovarian cancer, Health-related quality of life, Sleep disturbance, Fatigue, Symptom burden

## Abstract

**Background:**

Given that sleep disturbance and fatigue are pervasive among patients with advanced ovarian cancer (OC) and associated with poor health-related quality of life (HRQoL), several patient-reported outcome measures have been used to assess symptom burden. However, it remains unclear which patient-reported topic domains are most important to HRQoL for advanced OC patients. This study aimed to assess the domains of sleep disturbance and fatigue associated with poor HRQoL among patients with advanced OC across their treatment trajectory.

**Methodology:**

We conducted an online survey with adults diagnosed with Stage III or IV OC in the last three years. Patients reported clinical and sociodemographic characteristics, sleep disturbance and fatigue levels using Patient-Reported Outcomes Measurement Information System (PROMIS) short forms, and HRQoL using the CDC Healthy Days measure. Hierarchical regression models were used to assess how sleep disturbance and fatigue short-form items predicted poor HRQoL, specifically the number of days where poor health interfered with daily life and poor physical and mental health days (unhealthy days) in the past month, compared to clinical (time since treatment, treatments received, metastatic status) and sociodemographic (age, gender, marital status, employment) factors.

**Results:**

Among the 200 participants diagnosed with advanced OC (71.50% Stage III, 28.50% Stage IV; 33.50% receiving chemotherapy, 44.00% receiving maintenance therapy), sleep disturbance and fatigue were significantly associated with poor HRQoL. Compared to clinical and sociodemographic variables, sleep disturbance levels explained 17.00% more variance in the number of unhealthy days in the past month; fatigue levels explained 30.00% more variance in the number of unhealthy days. Among short form items, having difficulty falling asleep (β = 0.17, *p* = .03), fatigue interfering with physical function (β = 0.24, *p* < .01), and feeling run-down (β = 0.35, *p* < .01) were the strongest predictors of poor HRQoL.

**Conclusion:**

Patients with advanced OC reported significantly elevated levels of sleep disturbance and fatigue, which were associated with poor physical and mental health. Findings highlight the importance of screening for difficulty falling asleep, interference with physical function, and feeling run-down to connect patients with supportive care that may improve their HRQoL during and after treatment.

## Abstract (plain language summary)

Many people with ovarian cancer have trouble sleeping and experience fatigue, which make their quality of life worse. Healthcare providers have limited time with patients, so it can be difficult to identify and treat the symptoms that matter most to patients’ quality of life. In this study, we identified the issues that patients with ovarian cancer had with sleeping and fatigue that were most important to their quality of life by having them answer questions on a survey. We discovered that patients who said that they most often had difficulty falling asleep had a worse quality of life. Patients who said that fatigue often got in the way of doing physical activities and felt run-down also had poorer quality of life. This study demonstrated that there are three symptoms associated with difficulty sleeping and fatigue that are most important to ovarian cancer patients’ quality of life. During and after treatment, healthcare providers should ask people with ovarian cancer how much trouble they have falling asleep, how much fatigue gets in the way of doing physical activities, and how much they feel run-down. Asking these questions can help people get the support they need to improve their sleep and fatigue issues.

## Background

Sleep disturbance and fatigue are pervasive among cancer patients, including those with advanced ovarian cancer [1, 2]. Sleep disturbance includes difficulty falling and staying asleep, early waking, and poor sleep quality [3]. Fatigue includes persistent, intense, and distressing feelings of tiredness that impede physical function [4]. Fatigue and sleep disturbance are most intense during treatment with 92% of advanced OC patients reporting fatigue, and 80% reporting sleep disturbance [5]. These symptoms persist post-treatment into survivorship. Among survivors 12 months after completing treatment, 57–59% report moderate-to-severe fatigue [6, 7]; and 59% report moderate-to-severe insomnia [7]. Moderate sleep disturbance (63%) and severe fatigue (26%) persist among survivors five-years after completing treatment [8, 9]. Sleep disturbance and fatigue are associated with poor health-related quality of life (HRQoL) among advanced OC patients, including poor self-rated health, increased rates of depression, and increased reports of distress and anxiety [9–14]. Younger people with OC are at greater risk for sleep disturbance and fatigue and typically report poorer HRQoL compared to older patients [10, 13]. Sleep disturbance and fatigue limit patients’ abilities to engage in activities of daily life (ADLs), such as household chores and paid work, and reduced capacity for ADLs is associated with poor HRQoL [1, 10, 11]. Although identifying the causal direction and mechanisms in the relationship between symptom burden and HRQoL is challenging, previous research demonstrates that sleep disturbance and fatigue levels are independent predictors of HRQoL during and after treatment [9, 15, 16]. 

Given the prevalence of fatigue and sleep disturbance and its association with poor HRQoL, the National Comprehensive Cancer Center Network (NCCN) and the American Society of Clinical Oncology (ASCO) recommend screening and treating patients for sleep disturbance and fatigue using patient-reported questionnaires [4, 17]. However, there are significant barriers to effectively screening for sleep disturbance and fatigue. Healthcare providers cite time constraints and limited referral options as barriers to successfully screening and treating patients for fatigue [18–20]. Forty-three percent of cancer patients reported that their care team ever asked about fatigue, and among those who experienced severe fatigue, only 13% completed a screener about their symptoms [21].

There is little consensus as to the best screening tool for cancer patients or those with advanced OC [22]. A 2011 review identified 40 measurements for assessing fatigue in cancer patients [23] with varying degrees of psychometric validity and practicality in clinical settings. To measure fatigue in OC patients, studies have used the Functional Assessment of Chronic Illness Therapy – Fatigue (FACT-F), the Fatigue Questionnaire (FQ), the Multidimensional Fatigue Inventory (MFI-20), Profile of Mood States- Short form (POMS-SF), Fatigue Symptom Inventory (FSI), and the Patient-Reported Outcomes Measurement Information System (PROMIS) [8, 11, 13, 24–26].

While there are fewer tools available for screening for sleep disturbance and related conditions compared to fatigue [27], there is also little agreement as to the best screening tool for patients with OC. The Pittsburgh Sleep Quality Index (PSQI) and Insomnia Severity Index (ISI) have been utilized in studies of sleep disturbance and insomnia in patients with OC [9, 10, 12, 14, 28]. The diversity of PROMs available makes it challenging for clinicians to select screening tools.

Most sleep disturbance and fatigue PROMs are developed and validated based on adult populations, rather than disease-specific populations. However, there has been increasing attention to cancer-specific screening tools. The PROMIS measurement system includes a short form validated for cancer patients [29]. Scholars developed a PROM to assess OC patients’ unique symptom burden – the *MOST-S26* - for OC patients receiving chemotherapy, including an item for ‘trouble sleeping’ and ‘fatigue’ in the past three-four weeks [30]. This scale has been used in clinical studies in Australia [6, 9] but has yet to be applied widely in clinical and research settings in the U.S., where brief questionnaires like PROMIS short forms are increasingly common in clinical settings given their efficiency and strong psychometric properties [22, 31], and PROMIS sleep disturbance [32] and fatigue [31] measures have been validated for use among cancer patients.

Sleep disturbance and fatigue levels vary across the treatment trajectory for advanced OC patients, such that the sleep and fatigue experiences of post-chemotherapy patients may be distinct from those receiving maintenance therapy [26]. Sleep disturbance and fatigue are particularly intense during chemotherapy [5]. Although fatigue and sleep disturbance become milder during maintenance therapy and in survivorship, these symptoms have lasting impact on their HRQoL [7, 9, 33]. Given the challenges selecting and implementing tools to assess symptom burden relevant to OC patients, we assessed the domains of sleep disturbance and fatigue most associated with poor HRQoL for those with advanced OC.

## Methodology

### Survey instrument

The aim of this study was to assess the nature and extent of the burden of sleep disturbance and fatigue and their relationship with HRQoL among patients with Stage III and IV OC. We used *Qualtrics* to field an online survey with adults in the U.S. diagnosed with Stage III or IV ovarian cancer within the last three years. The survey instrument was designed to assess patients’ symptom burden before, during, and after treatment for OC, in addition to coping strategies and patient-provider communication. Survey content was developed based on in-depth interviews with Stage III and IV OC patients about their experiences with sleep disturbance and fatigue at different points in their treatment trajectory [24].

The primary outcome, HRQoL, was assessed using the CDC Healthy Days measure (CDC-HRQoL-4) [34]. Participants rated their current health (poor, fair, very good, good, excellent), the number of days in the last month in which their physical health was not good (0–30), the number of days in which their mental health was not good (0–30), and the number of days in which poor physical or mental health interfered with their ability to do usual activities. The number of poor physical and mental health days are summed into an index of “unhealthy days” and subtracted from 30 to ascertain the number of healthy days in a month (0–30). The CDC-HRQoL-4 been used since 1993 in the Behavioral Risk Factor State Survey as an efficient measure of HRQoL and has strong construct validity compared to other instruments of HRQoL [35, 36].

The primary predictors of HRQoL, sleep disturbance and fatigue, were measured using the Patient-reported Outcomes Measurement Information System (PROMIS) Short Forms (SFs): Sleep Disturbance – SF 8b (PROMIS SD-SF-8b) and Fatigue – SF 8a (PROMIS F-SF-8a). Participants rated the frequency (*1 = Never; 5 = Always*) and intensity (*1 = Not at all; 5 = Very much*) with which they experienced each symptom on the respective scales during the past 7 days. Short form scores were converted to standardized T-scores based on the general population (Mean = 50; SD = 10). Higher T-scores indicate more symptom burden with +/-3 being clinically significant [37]. Responses to individual sleep disturbance and fatigue short-form items were dichotomized to create a binary measure of the most intense symptom burden (e.g., Quite a bit/Very much vs. sometimes/a little bit/not at all; Always/often vs. sometimes/rarely/never) to assess how intense symptom burden was associated with poor HRQoL.

### Survey execution

**Fig. 1:**
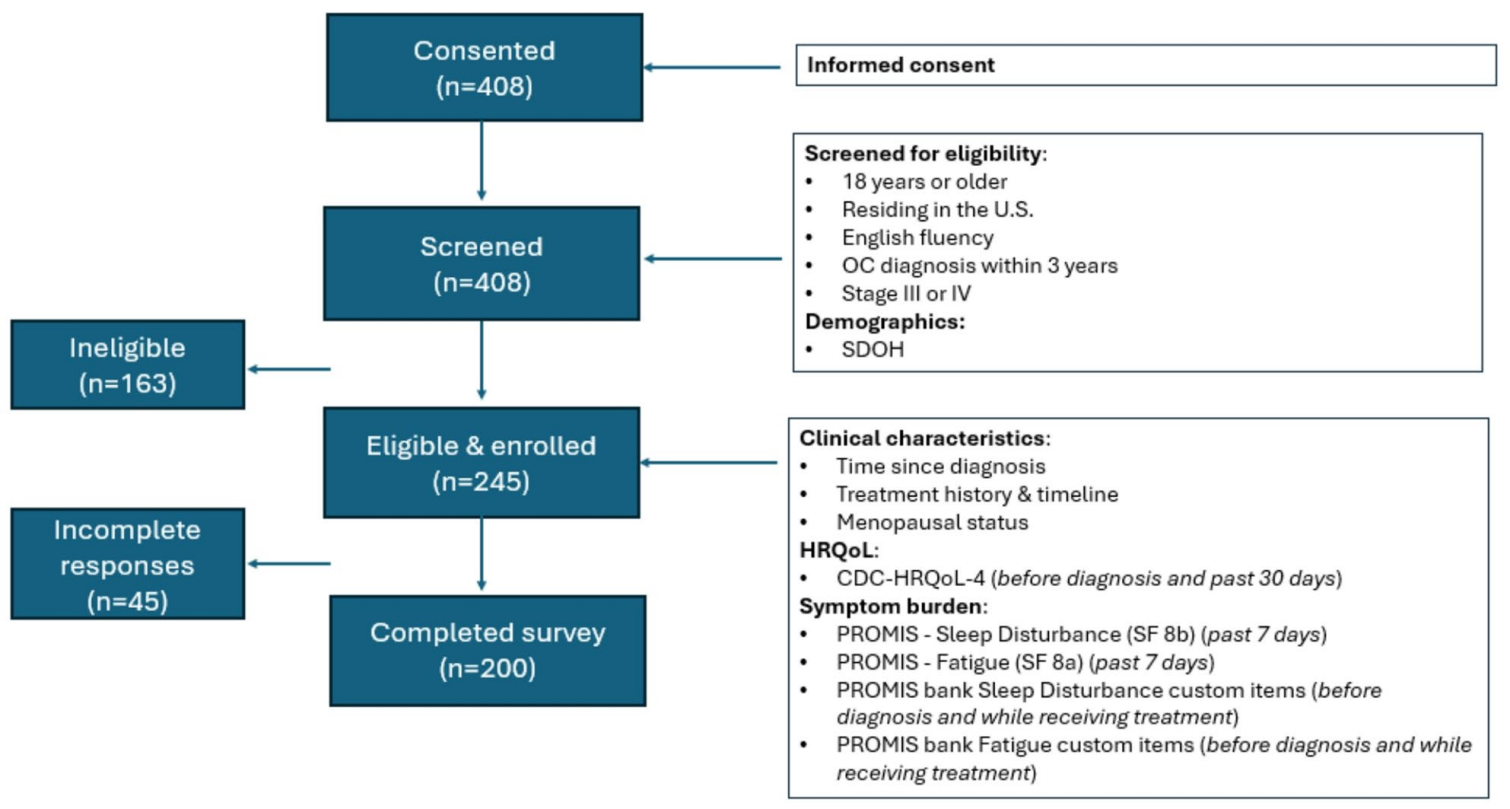
Study flow chart

Participants were recruited from Cancer Support Community’s 196 network partners and nine advocacy organizations. Participants were asked to review the consent form and provide electronic consent before completing the survey. Participants received a $35 e-gift card for completing the survey. Data quality checks were implemented and responses removed if two of five criteria were met: (1) suspicious survey link activity; (2) failed ReCAPTCHA; (3) accessing the survey from the same IP address multiple times; (4) accessing the survey from an international IP address; and (5) straight lining and illogical responses. After removing these cases, 163 were ineligible because they did not meet study criteria, 45 were eligible but did not complete the survey, and 200 were eligible and completed the survey (*See* Fig. 1 *for Study Design*). Responses were first reviewed by the *Qualtrics* bot detection algorithm. Then, one study team member (MG) checked each response for the five criteria above. Responses that met two or more of the criteria above were reviewed by a second team member (AN). A third team member (EF) reviewed inconsistencies in data quality checks before establishing the analytic dataset (*n* = 200).

Data were cleaned and analyzed using *IBM SPSS Statistics* version 29. In addition to descriptive statistics, t-tests, and bivariate Pearson correlations, we employed four hierarchical regression models to assess the relationship between poor HRQoL, sleep disturbance, and fatigue. As implemented here, hierarchical regression involves the sequential addition of variables in blocks to determine which variables best explain the variance in the outcome variable. Variables were entered into the hierarchical regression models in theoretically- and temporally- informed blocks to examine the incremental contribution of each domain in explaining HRQoL. Person-level demographic factors were entered into the first block as covariates: age (continuous), married (yes/no), living alone (yes/no), and employment status (employed part or full time, not employed due to disability, not employed due to retirement or other reason). Clinical history variables were added in the second block to account for more proximal factors that may moderate the relationship between demographics and HRQoL: years since diagnosis (continuous), metastatic status (ever metastatic vs. not), treatment history (binary yes/no for surgery, chemotherapy, and poly-ADP ribose inhibitor (PARPi) maintenance therapy). Finally, symptomatology variables were entered in the third block, as these represent the potentially modifiable factors hypothesized to have the strongest direct relationship with HRQoL. For two of the models, block three contained the eight binary items for PROMIS Sleep Disturbance – SF-8b, while the other two contained the items for PROMIS Fatigue – SF-8a. Results were considered statistically significant at the *p* < .05 level and trending at the *p* < .10 level. This approach provides insight into the predictive validity of the individual sleep disturbance and fatigue items compared to demographic and clinical characteristics [38].

## Results

### Sample characteristics

200 U.S. adults with Stages III (71.50%) and IV (28.50%) ovarian cancer completed the survey between August and November 2023 (Table 1). The average age was 56.20 years-old (range: 25 to 84). Most participants were non-Hispanic White (82.50%) with a bachelor’s degree or higher (67.50%). Thirty-seven percent were working, 29.00% were retired, and 22.00% were not working due to disability. Most (69.50%) were married or in a long-term partnership, and 15.50% were currently living alone. Half (51.00%) of participants were within 2 years of diagnosis, and 31.00% had experienced a recurrence since diagnosis. Most were receiving active treatment with chemotherapy (33.50%) or maintenance therapy (44.00%) with PARPi, and the most common treatment combination being surgery, chemotherapy, PARPi maintenance therapy, and other targeted therapies (44.00%).

**Table 1:** Sample Characteristics (*N* = 200)

	*n* (%)
Age, mean (SD)	56.20 (12.60)
Age, min-max	25–84
Gender Identity, n (%)	
* Woman*	199 (99.50)
* Non-binary*	1 (0.50)
Race, n (%)	
* American Indian/Alaska Native*	1 (0.50)
* Asian*	2 (1.00)
* Black/African American*	13 (6.50)
* Hispanic/Latino*	10 (5.00)
* White (non-Hispanic)*	165 (82.50)
* Multiple races*	7 (3.50)
*Other / prefer not to share*	2 (1.00)
Education, n (%)	
* Trade school*	6 (3.00)
* High school or equivalent*	9 (4.50)
* Some college but no degree*	31 (15.50)
* Associate’s degree*	19 (9.50)
* Bachelor’s degree*	64 (32.00)
*Advanced degree*	71 (35.50)
Work status, n (%)	
* Working for wages*	74 (37.00)
* Retired*	58 (29.00)
* Not working due to disability*	44 (22.00)
* Not working for other reason*	22 (11.00)
*Prefer not to share*	2 (1.00)
Marital Status, n (%)	
* Married / Partnered*	139 (69.50)
*Single / Divorced / Widowed*	61 (30.50)
Living alone, n (%)	31 (15.50)
Stage at diagnosis, n (%)	
* Stage III*	143 (71.50)
* Stage IV*	57 (28.50)
Time since diagnosis, mean (SD) in years	1.46 (1.00)
Recurrence, n (%)	62 (31.00)
Treatment Status, n (%)	
* Completed treatment*	29 (14.50)
* Receiving active treatment*	67 (33.50)
* Receiving maintenance therapy*	88 (44.00)
* Other / unsure*	16 (8.00)
Treatment Groups, n (%)	
* Surgery & chemotherapy*	43 (21.50)
* Surgery*,* chemotherapy*,* & PARPi + others*	88 (44.00)
* All other combinations; no PARPi*	69 (34.50)
Sleep Disturbance T Score, mean (SD)	56.93 (5.40)
Fatigue T Score, mean (SD)	60.88 (9.13)
Unhealthy days in the past month, mean (SD)	14.40 (11.24)
* Poor physical health days*,* mean (SD)*	8.44 (8.74)
* Poor mental health days*,* mean (SD)*	8.54 (8.97)
Days with poor health interference, mean (SD)	8.24 (9.29)
Fair/poor self-rated health, n (%)	60 (30.00)

#### Descriptive results

Most (59.00%) participants reported significantly higher rates of fatigue compared to the general population, and many (39.00%) reported higher rates of sleep disturbance than the general population. The mean T-score for sleep disturbance (56.93 [5.40]) was clinically and statistically elevated compared to the general population mean of 50 (*t* = 18.06; *p* < .01), and the mean T-score for fatigue was statistically and clinically elevated (60.88 [9.13]) compared to general population mean of 50 (*t* = 16.85, *p* < .01).

For the eight binary sleep disturbance items, endorsement ranged from 37.00% to 57.50%, with 37.00% of participants indicating their sleep was poor or very poor, and 57.50% of participants saying that their sleep was not at all or a little bit refreshing. For fatigue, endorsement ranged from 43.50% to 61.50%, with 43.50% of participants quite a bit to very much agreeing that they have trouble starting things because they are tired, and 43.50% quite a bit to very much agreeing that fatigue interfered with their physical functioning. For the most endorsed item, 61.50% indicated they often to always have to push themselves to get things done because of their fatigue (Table 2).

**Table 2:** PROMIS Sleep Disturbance and Fatigue Item-level Responses (*N* = 200)

Short Form Item	Response categories	*n* (%)
**Sleep Disturbance (SF-8b)**		
* My sleep was restless*	*Quite a bit –* *Very much*	100 (50.00%)
* I was satisfied with my sleep*	*Not at all –* *A little bit*	100 (50.00%)
* My sleep was refreshing*	*Not at all –* *A little bit*	115 (57.50%)
* I had difficulty falling asleep*	*Quite a bit –* *Very much*	78 (39.00%)
* I had trouble staying asleep*	*Often - Always*	111 (55.50%)
* I had trouble sleeping*	*Often - Always*	92 (46.00%)
* I got enough sleep*	*Never - Rarely*	85 (42.50%)
* My sleep quality is…*	*Poor - Very Poor*	74 (37.00%)
**Fatigue (SF-8a)**		
* I feel fatigued*	*Quite a bit –* *Very much*	115 (57.50%)
* I have trouble starting things because I am tired*	*Quite a bit –* *Very much*	87 (43.50%)
* How run down do you feel on average?*	*Quite a bit –* *Very much*	100 (50.00%)
* How fatigued are you on average?*	*Quite a bit –* *Very much*	103 (51.50%)
* How much were you bothered by your fatigue on average?*	*Quite a bit –* *Very much*	105 (52.50%)
* To what degree did your fatigue interfere with your physical functioning?*	*Quite a bit –* *Very much*	87 (43.50%)
* How often do you have to push yourself to get things done because of your fatigue?*	*Often - Always*	123 (61.50%)
* How often do you have trouble finishing things because of your fatigue?*	*Often - Always*	96 (48.00%)

### Health-related quality of life

Participants most frequently rated their health as “good” (42.00%), with 1.00% reporting excellent health, 28.00% reporting very good health, 24.00% fair health, and 6.00% poor health. Participants reported an average of 8.24 days of interference with daily living (SD = 9.29; range = 0–30), 8.44 days of poor physical health days (SD = 8.74; range 0–30), and 8.54 days of poor mental health days (SD = 8.97; range = 0–30). The upper quartile for each of the unhealthy day measures fell at 14 or 15 days, similar to previous findings about the upper quartile of unhealthy days in the general population [34, 35]. The composite measure for unhealthy physical and mental days resulted in 25.00% of the sample having the highest possible value (30) with an average of 14.40 unhealthy days (SD = 11.24) (See Table 1).

#### Bivariate results

Higher sleep disturbance and fatigue levels were significantly associated with poor HRQoL. Higher sleep disturbance T-scores, indicating more symptom burden, were significantly associated with the number of poor mental health days in the past month (*r* = .46, *p* <. 01). High endorsement of sleep disturbance symptom burden items was also significantly associated with poor HRQoL (Table 3). Higher fatigue scores were significantly correlated with poor HRQoL measures (Table 4), including the number of poor physical health days in the past month (*r* = .53, *p* < .01), and this association was significant for high endorsement of symptom response items as well.

**Table 3:** Correlation of Health-related Quality of Life with Sleep Disturbance Measures

HRQoL - CDC HRQoL − 4 (past 30 days)	1	2	3	4	5	6	7	8	9	10	11	12	13
*1. Self-rated health*	-												
*2. Days when poor health limited activities*	0.42***	-											
*3. Poor physical health days*	0.49**	0.76***	-										
*4. Poor mental health days*	0.36***	0.55***	0.54***	-									
**Mean PROMIS T-scores** (past 7 days)													
*5. Sleep Disturbance T-score*	0.37***	0.33***	0.30***	0.46***	-								
**Sleep Disturbance SF 8b Items** (past 7 days)													
*6. My sleep was restless*	0.20**	0.25***	0.20**	0.36***	0.81***	-							
*7. I was satisfied with my sleep*	-0.30***	-0.26***	-0.21**	-0.35***	-0.79***	-0.55***	-						
*8. My sleep was refreshing*	-0.35***	-0.23***	-0.24***	-0.38***	-0.76***	-0.51***	0.70***	-					
*9. I had difficulty falling asleep*	0.27***	0.28***	0.28***	0.36***	0.61***	0.45***	-0.38***	-0.34***	-				
*10. I had trouble staying asleep*	0.19**	0.18**	0.18*	0.28***	0.71***	0.66***	-0.44***	-0.40***	0.19**	-			
*11. I had trouble sleeping*	0.27***	0.31***	0.31***	0.41***	0.86***	0.72***	-0.59***	-0.56***	0.48***	0.75***	-		
*12. I got enough sleep*	-0.32***	-0.25***	-0.23***	-0.35***	-0.80***	-0.51***	0.64***	0.66***	-0.38***	-0.51***	-0.64***	-	
*13. My sleep quality was…*	0.41***	0.29***	0.23***	0.37***	0.86***	0.66***	-0.68***	-0.65***	0.40***	0.58***	0.69***	-0.74***	-

Note: *p<.05; **p<.01; ***p<.001

**Table 4:** Correlation of Health-related Quality of Life with Fatigue Measures

HRQoL - CDC HRQoL − 4 (past 30 days)	1	2	3	4	5	6	7	8	9	10	11	12	13
*1. Self-rated health*	-												
*2. Days when poor health limited activities*	0.42***	-											
*3. Poor physical health days*	0.49**	0.76***	-										
*4. Poor mental health days*	0.36***	0.55***	0.54***	-									
**Mean PROMIS T-scores** (past 7 days)													
*5. Fatigue T-Score*	0.46***	0.52***	0.53***	0.44***	-								
**Fatigue SF 8a Items** (past 7 days)													
*6. I feel fatigued*	0.39***	0.42***	0.42***	0.37***	0.89***	-							
*7. I have trouble starting things because I am tired*	0.44***	0.48***	0.45***	0.43***	0.87***	0.76***	-						
*8. How run-down did you feel on average?*	0.41***	0.50***	0.54***	0.42***	0.90***	0.82***	0.74**	-					
*9. How fatigued were you on average?*	0.40***	0.48***	0.48***	0.40***	0.91***	0.86***	0.76***	0.91***	-				
*10. How much were you bothered by your fatigue on average?*	0.35***	0.41***	0.47***	0.42***	0.86***	0.75***	0.70***	0.77***	0.78***	-			
*11. To what degree did your fatigue interfere with your physical functioning?*	0.39***	0.54***	0.53***	0.40***	0.88***	0.76***	0.74***	0.78***	0.78***	0.77***	-		
*12. How often did you have to push yourself to get things done because of your fatigue?*	0.38***	0.40***	0.38***	0.39***	0.87***	0.72***	0.72***	0.72***	0.73***	0.73***	0.76***	-	
*13. How often did you have trouble finishing things because of your fatigue?*	0.37***	0.48***	0.44***	0.37***	0.85***	0.67***	0.78***	0.71***	0.70***	0.67***	0.74***	0.79***	-

Note: *p<.05; **p<.01; ***p<.001

#### Multivariate results

##### Sleep disturbance and HRQoL

Being unemployed due to disability was the only significant predictor of the number of days in which poor health interfered with daily life in Block 1 and was associated with higher daily interference (β = 0.38, *p* < .01). Retired/other unemployment was also significant, but had a weaker association (β = 0.17, *p* = .05). In Block 2, the only trending clinical predictor was time since diagnosis, which exhibited a negative association (β = -0.14, *p* = .05), such that more time since diagnosis resulted in less daily interference. The individual sleep disturbance items were not significant predictors, yet the sleep disturbance block accounted for a significant amount of variance explained in the full model, with all blocks and predictors accounting for 29.00% of the variance in interference with daily living. Moreover, the addition of sleep disturbance short-form items to the model explains 9.50% more variance in the number of unhealthy days than clinical and sociodemographic variables (Table 5).

Unemployment due to disability was also associated with the number of unhealthy days in the past month (β = 0.18, *p* = .01), and there were no significant clinical predictors in Block 2. One significant and one trending sleep disturbance item emerged in Block 3: difficulty falling asleep (β = 0.17, *p* = .03) and poor sleep quality (β = 0.19, *p* = .06). All blocks and predictor variables accounted for 34.00% of the variance in unhealthy days. The addition of sleep disturbance short-form items to the model explains 17.00% more variance in the number of unhealthy days than clinical and sociodemographic variables. Thus, sleep disturbance, particularly difficulty falling asleep, is a strong predictor of poor HRQoL, beyond clinical characteristics and sociodemographic background.

**Table 5:** Hierarchical Regression Models of Sleep Disturbance-related Factors of Poor HRQoL

	Interference with daily living (*N* = 196)							Unhealthy days (*N* = 195)						
						95% CI EXP(B)							95% CI EXP(B)	
	B	SE	Beta	t	Sig.	Lower	Upper	B	SE	Beta	t	Sig.	Lower	Upper
Age	-0.02	0.06	-0.03	-0.36	0.72	-0.14	0.10	-0.03	0.07	-0.04	-0.47	0.64	-0.17	0.11
Married	0.32	1.57	0.02	0.20	0.84	-2.78	3.42	-0.06	1.84	-0.00	-0.03	0.97	-3.68	3.56
Live Alone	0.67	2.06	0.03	0.33	0.75	-3.40	4.74	0.73	2.41	0.02	0.30	0.76	-4.03	5.49
Not employed retire/other	**3.16**	**1.57**	**0.17**	**2.01**	**0.05**	**0.06**	**6.27**	-1.33	1.85	-0.06	-0.72	0.47	-4.97	2.32
Not employed disability	**8.41**	**1.65**	**0.38**	**5.11**	**< 0.01**	**5.16**	**11.66**	**4.93**	**1.92**	**0.18**	**2.56**	**0.01**	**1.13**	**8.72**
Block Summary	R^2^=0.16; Adjusted R^2^ = 0.13							R^2^=0.16; Adjusted R^2^ = 0.13						
	F change = 6.98***							F change = 6.96***						
Years since diagnosis	**-1.32**	**0.66**	**-0.14**	**-2.02**	**0.05**	**-2.62**	**-0.03**	-1.09	0.77	-0.10	-1.42	0.16	-2.60	0.43
Metastatic	1.47	1.35	0.07	1.09	0.28	-1.19	4.12	0.23	1.57	0.01	0.15	0.88	-2.88	3.34
Past surgery	-3.17	2.37	-0.09	-1.34	0.18	-7.85	1.52	-1.72	2.77	-0.04	-0.62	0.54	-7.20	3.75
Past/current chemotherapy	2.07	3.30	0.04	0.63	0.53	-4.44	8.59	-4.04	3.86	-0.07	-1.05	0.30	-11.65	3.57
Past/current PARPi	0.24	1.35	0.01	0.18	0.86	-2.42	2.90	-1.28	1.58	-0.06	-0.81	0.42	-4.40	1.84
Block Summary	R^2^=0.19; Adjusted R^2^ = 0.15							R^2^=0.18; Adjusted R^2^ = 0.13						
	R^2^ change = 0.03; F change = 1.58(ns)							R^2^ change = 0.02; F change = 0.96(ns)						
*My sleep was restless*	0.21	1.62	0.01	0.13	0.90	-2.99	3.41	-0.28	1.89	-0.01	-0.15	0.88	-4.02	3.46
*I was satisfied with my sleep*	2.08	1.75	0.11	1.18	0.24	-1.39	5.54	0.69	2.05	0.03	0.34	0.74	-3.35	4.73
*My sleep was refreshing*	0.08	1.61	0.01	0.05	0.96	-3.10	3.26	1.63	1.89	0.07	0.86	0.39	-2.10	5.36
*I had difficulty falling asleep*	1.39	1.46	0.07	0.96	0.34	-1.48	4.27	**3.80**	**1.70**	**0.17**	**2.23**	**0.03**	**0.44**	**7.16**
*I had trouble staying asleep*	-0.63	1.60	-0.03	-0.40	0.69	-3.80	2.53	0.39	1.88	0.02	0.21	0.84	-3.31	4.09
*I had trouble sleeping*	1.13	1.88	0.06	0.60	0.55	-2.57	4.83	2.70	2.19	0.12	1.23	0.22	-1.62	7.03
*I got enough sleep*	0.44	1.66	0.02	0.26	0.79	-2.83	3.71	0.15	1.97	0.01	0.08	0.94	-3.73	4.04
*My sleep quality is poor/very poor*	3.10	1.89	0.16	1.64	0.10	-0.63	6.83	**4.28**	**2.21**	**0.19**	**1.93**	**0.06**	**-0.09**	**8.64**
Block Summary	R^2^=0.29; Adjusted R^2^ = 0.21							R^2^=0.34; Adjusted R^2^ = 0.28						
	R^2^ change = 0.10; F change = 2.95**							R^2^ change = 0.17; F change = 5.63***						
**Full Model**	F = 3.92*							F = 5.14***						

*p<.05; **p<.01; ***p<.001; CI=Confidence Interval

##### Fatigue and HRQoL

Consistent with models of sleep disturbance and poor HRQoL, unemployment due to disability was significantly associated with daily interference (β = 0.33, *p* < .01) in Block 1 and years since diagnosis was trending (β = -0.12, *p* = .06) in Block 2. Fatigue interfering with physical functioning was a significant predictor (β = 0.24, *p* < .01) and trouble finishing tasks due to fatigue was trending (β = 0.15, *p* = .08) in Block 3. All predictors accounted for nearly 43% of the variance in interference with daily living, with fatigue items added in Block 3 exhibiting the greatest amount of variance explained (26.00%). (Table 6)

**Table 6:** Hierarchical Regression Models of Fatigue-Related Factors of Poor HRQoL

	Interference with daily living (*N* = 198)							Unhealthy days (*N* = 197)						
						95% CI EXP(B)							95% CI EXP(B)	
	B	SE	Beta	t	Sig.	Lower	Upper	B	SE	Beta	t	Sig.	Lower	Upper
Age	-0.07	0.05	-0.09	-1.24	0.22	-0.17	0.04	**-0.14**	**0.06**	**-0.15**	**-2.24**	**0.03**	**-0.26**	**-0.02**
Married	-0.20	1.40	-0.01	-0.14	0.89	-2.96	2.56	-0.2	1.64	-0.01	-0.12	0.90	-3.43	3.03
Live Alone	1.15	1.87	0.04	0.62	0.54	-2.53	4.83	1.63	2.18	0.05	0.75	0.46	-2.68	5.93
Not employed retire/other	2.20	1.44	0.12	1.53	0.13	-0.63	5.03	-1.99	1.69	-0.09	-1.18	0.24	-5.33	1.34
Not employed disability	**7.29**	**1.54**	**0.33**	**4.72**	**< 0.01**	**4.24**	**10.34**	**4.27**	**1.81**	**0.16**	**2.37**	**0.02**	**0.71**	**7.84**
Block Summary	R^2^=0.14; Adjusted R^2^ =0.12							R^2^=0.15; Adjusted R^2^ =0.13						
	F change = 6.43***							F change = 6.94						
Years since diagnosis	**-1.10**	**0.58**	**-0.12**	**-1.91**	**0.06**	**-2.23**	**0.04**	-0.96	0.67	-0.09	-1.43	0.15	-2.29	0.37
Metastatic	1.82	1.20	0.09	1.52	0.13	-0.55	4.20	0.97	1.41	0.04	0.69	0.49	-1.81	3.75
Past surgery	-3.58	2.13	-0.10	-1.68	0.10	-7.79	0.64	-1.85	2.50	-0.04	-0.74	0.46	-6.78	3.08
Past/current chemotherapy	2.70	3.04	0.05	0.89	0.38	-3.30	8.70	-2.09	3.55	-0.04	-0.59	0.56	-9.11	4.92
Past/current PARPi	-0.16	1.17	-0.01	-0.14	0.89	-2.48	2.15	-1.68	1.38	-0.07	-1.22	0.22	-4.39	1.03
Block Summary	R^2^=0.17; Adjusted R^2^ = 0.13							R^2^=0.18; Adjusted R^2^ = 0.13						
	R^2^ change = 0.03; F change = 1.38(ns)							R^2^ change = 0.02; F change = 0.98(ns)						
*I feel fatigued*	-0.26	1.77	-0.01	-0.15	0.88	-3.76	3.24	-0.74	2.07	-0.03	-0.36	0.72	-4.83	3.35
…*trouble starting things because I am tired*	1.20	1.59	0.06	0.75	0.45	-1.94	4.34	2.04	1.86	0.09	1.10	0.27	-1.63	5.70
*How run down do you feel on average*	3.02	2.18	0.16	1.38	0.17	-1.29	7.32	**7.84**	**2.55**	**0.35**	**3.07**	**< 0.01**	**2.80**	**12.87**
*How fatigued are you on average*	1.69	2.33	0.09	0.72	0.47	-2.92	6.29	0.54	2.73	0.02	0.20	0.84	-4.84	5.92
*bothered by your fatigue …*	0.51	1.54	0.03	0.33	0.74	-2.53	3.54	2.71	1.80	0.12	1.50	0.13	-0.84	6.27
… *interfere with your physical functioning*	**4.47**	**1.54**	**0.24**	**2.91**	**< 0.01**	**1.44**	**7.50**	1.79	1.80	0.08	0.99	0.32	-1.75	5.33
*push yourself to get things done…*	-1.99	1.53	-0.10	-1.30	0.20	-5.01	1.04	-1.45	1.79	-0.06	-0.81	0.42	-4.98	2.09
*have trouble finishing things…*	**2.71**	**1.54**	**0.15**	**1.76**	**0.08**	**-0.32**	**5.74**	1.86	1.80	0.08	1.04	0.30	-1.68	5.41
Block Summary	R^2^=0.43; Adjusted R^2^ = 0.38							R^2^=0.47; Adjusted R^2^ = 0.42						
	R^2^ change = 0.26; F change = 10.26***							R^2^ change = 0.30; F change = 12.37***						
**Full Model**	F = 7.62***							F = 8.78***						

*p<.05; **p<.01; ***p<.001; CI=Confidence Interval

Unemployment due to disability is also a significant predictor of unhealthy days (β = 0.16, *p* = .02), along with younger age (β = -0.15, *p* = .03). Among fatigue items, feeling run down was strongly associated with more unhealthy days (β = 0.35, *p* < .01). While no other significant items emerged, the block explained the largest amount of variance in unhealthy days (30.00%) with the full model having 47.00% variance explained. Fatigue levels are the most significant predictors of HRQoL compared to clinical and sociodemographic background, illustrating how symptom burden negatively impacts patients’ quality of life.

## Discussion

This study assessed the sleep- and fatigue-related domains most associated with poor HRQoL among patients with advanced OC across the treatment trajectory. Findings suggest that clinically and statistically elevated levels of sleep disturbance and fatigue are prevalent among patients with advanced OC, and this high symptom burden is associated with poor mental health, poor physical health, and poor health interfering with daily life. In models of the relationship between sleep disturbance and poor HRQoL, difficulty falling asleep and poor sleep quality were associated with poor HRQoL, along with not working due to disability and being recently diagnosed. Feeling run-down and fatigue interfering with physical function were most associated with poor HRQoL, in addition to being younger, recently diagnosed, and not working due to disability. Across all models, levels of sleep disturbance and fatigue better accounted for the variation in patients’ HRQoL than sociodemographic and clinical characteristics, including treatment history and time since diagnosis.

Findings are consistent with previous studies demonstrating the prevalence of sleep disturbance and fatigue burden among patients with advanced OC [10, 12, 26, 39]. Approximately 56% of the sample reported statistically significant levels of fatigue, which is similar to symptom prevalence of patients within one year of completing treatment in previous studies [7]. Elevated levels of sleep disturbance have also been documented by cross-sectional studies of OC patients, including patients experiencing recurrence [5] and survivors five years post-treatment [8]. Sleep disturbance and fatigue have been linked to poor HRQoL in several studies [9–12, 28]. Clinical characteristics, including metastatic status, treatment status, and treatments received were not significant predictors of HRQoL compared to symptom burden. The impact of disease and treatment-related factors may be mediated through symptom burden. Moreover, this finding is consistent with previous research that symptom burden and poor HRQoL can persist into survivorship [8, 13, 40].

Results illustrate the importance of granular, symptom-specific assessments in understanding the relationship between symptom burden and HRQoL. The addition of symptom-related variables in Block 3 consistently improved model fit and explained a notable proportion of the variance in HRQoL, indicating the critical role of sleep- and fatigue-related symptoms in physical and mental well-being in patients with OC. Patients with advanced OC should be assessed for sleep disturbance and fatigue symptom burden across their treatment trajectory [26, 28]. Given that fatigue explained a larger proportion of variance in HRQoL compared to sleep disturbance, increased screening and connections with support for fatigue is critical [33]. Exercise programs and acceptance and commitment therapy have been shown to help manage fatigue among ovarian cancer patients [41, 42], as well as cognitive behavioral therapy and pharmacological interventions to mitigate sleep disturbance [43].

Finally, results underscore the role that emotional distress plays in the relationship between sleep disturbance, fatigue, and poor HRQoL. The sleep disturbance domains most associated with poor HRQoL were difficulty falling asleep and poor sleep quality, which are associated with anxiety [28]. Anxiety about recurrence may make it difficult to fall and stay asleep. Feeling run-down had the largest effect size of any predictor across models of fatigue and HRQoL. Feeling run-down is a hallmark of depression, which may help explain the relationship between fatigue and HRQoL [8, 10]. Given that fatigue and depression are intertwined among patients with OC [8], future research should assess how depression mediates the relationship between fatigue and HRQoL or exacerbates fatigue and decreases HRQoL.

Findings are consistent with previous studies which illustrate that younger patients and those unemployed or not working due to disability are most impacted by fatigue and sleep disturbance and often report poorer HRQoL overall [10, 13, 44, 45]. Among adolescent and young adult cancer patients, HRQoL is a primary concern [46, 47]. Younger patients may be managing competing responsibilities compared to older patients, including work and caregiving [48], which may contribute to their symptom burden and poorer HRQoL. Younger patients may require additional screening and support to manage symptoms and improve HRQoL. Not working due to disability is a robust predictor of poor HRQoL across all models, which is consistent with previous findings that the ability to work is associated with better HRQoL [44, 49]. Unemployed patients are more likely to report severe fatigue [49], and symptoms burden may make it difficult to return to work for those on disability leave, which underscores the need for supportive interventions to help patients manage symptoms and return to work. Conversely, the financial and emotional stressors of not working have been found to predict high symptom burden and poor HRQoL [49, 50]. Determining the causality in this relationship is difficult with cross-sectional data. Future research should assess the roles that fatigue and sleep disturbance play in limiting the ability to work and impacting HRQoL, in addition to the critical role that work status plays as a social determinant of health.

### Limitations of the study

Although this study provides valuable insights into the factors predicting poor HRQoL for patients with advanced OC, there are key limitations. This study did not collect participants’ medical records, so diagnoses cannot be validated against self-reports. However, participants were recruited via reputable psychosocial provider networks and advocacy partners to ensure that participants had been diagnosed with OC. Additionally, the convenience sample method and retrospective reporting may result in response and recall bias, skewing the results for respondents with the most intense symptom burden and poor HRQoL. However, the prevalence and intensity of sleep disturbance and fatigue are consistent with prior studies of patients with OC [6, 7], as well as the association between sleep, fatigue, and HRQoL [8, 10]. The results of this study may not be generalizable to all advanced OC patients because the sample is disproportionately White and highly-educated compared to the patient population. Given that most clinical characteristics were not significant predictors of QoL, the study’s modest sample size may have limited the statistical power to identify significant differences among clinical subgroups. Future research should assess predictors of QoL using more clinically and demographically diverse samples to better identify subgroup differences and disparities. Finally, the sample size provides sufficient statistical power to assess the study’s primary aims, but not enough power to conduct mediation analyses of covariates with sleep disturbance and fatigue symptoms. Future studies should leverage existing representative datasets of patients with OC to assess the factors mediating the relationship between symptom burden and HRQoL.

The survey was designed based on previous literature and data gathered from semi-structured interviews with advanced OC patients in a previous phase of this study [24]. The measures were selected based on these data to reflect the intensity and variation in symptom burden that patients reported in interviews. However, there are a multitude of measures for HRQoL, sleep disturbance, and fatigue. The European Organization of Research and Treatment of Cancer (EORTC) QoL (Quality of life) questionnaire is commonly used to assess HRQoL in cancer patients, including those with OC [51, 52]. However, this questionnaire may overburden patients taking the survey compared to the 4-item CDC Healthy Days measure. The FACT-O scale was developed specifically for assessing symptom burden in OC patients, but items are not specific to sleep disturbance and fatigue [53]. Future studies should assess the relationship between sleep and fatigue with different measures of HRQoL. To assess sleep disturbance and fatigue levels, we employed two validated short forms from the PROMIS bank that have been increasingly used in clinical settings [31]. Employing other PROMs of disordered sleep and fatigue may have yielded different results, which provides the opportunity for comparing these tools in predicting HRQoL.

## Conclusions

High symptom burden of sleep disturbance and fatigue is prevalent among patients with advanced OC, and these symptoms are significant predictors of poor HRQoL among patients with various metastatic statuses, treatment histories, and sociodemographic backgrounds. Symptom burden is a strong predictor of HRQoL, and thus, patients and survivors should be adequately screened across their treatment trajectory and beyond. Screening patients for sleep disturbance and fatigue is critical to connect them with treatment and supportive care, which may improve patients’ HRQoL. Although the proliferation of PROMs provides clinicians multiple tools to screen for - and potentially treat - symptom burden, it can be difficult to ascertain what PROMs are most effective and meaningful to patients, given providers’ limited time and resources. This study’s findings suggest that clinicians should focus their screening among patients with advanced OC on the sleep and fatigue domains most important to HRQoL: difficulty falling asleep, fatigue interfering with physical function, and feeling run-down, which were the most significant predictors of poor HRQoL. Providers should ask patients’ responses to these specific items to identify those in need of further symptom management.

## Data Availability

The datasets generated during the current study are not publicly available due to their proprietary nature.

## Abbreviations

ADL(s): Activities of Daily Life
ASCO: American Society of Clinical Oncology
BRFSS: Behavioral Risk Factor Surveillance System
CDC: Centers for Disease Control
FACIT-F: Functional Assessment of Chronic Illness Therapy - Fatigue
FSI: Fatigue Symptom Inventory
HRQoL: Health-related quality of life
ISI: Insomnia Severity Index
MFI: Multidimensional Fatigue Inventory
NCCN: National Comprehensive Cancer Network
OC: Ovarian cancer
POMS-F: Profile of Mood States - Fatigue
PROM(s): Patient-reported outcome measures
PROMIS: Patient-Reported Outcomes Measurement Information System
PSQI: Pittsburgh Sleep Quality Index
SF: Short form
